# Supplementary figures and images for: Perceptions of professional soccer coaches, support staff and players toward virtual reality and the factors that modify their intention to use it
Source: PLoS One. 2021 Dec 30;16(12):e0261378. doi: 10.1371/journal.pone.0261378 (PMC8717979; doi:10.1371/journal.pone.0261378)

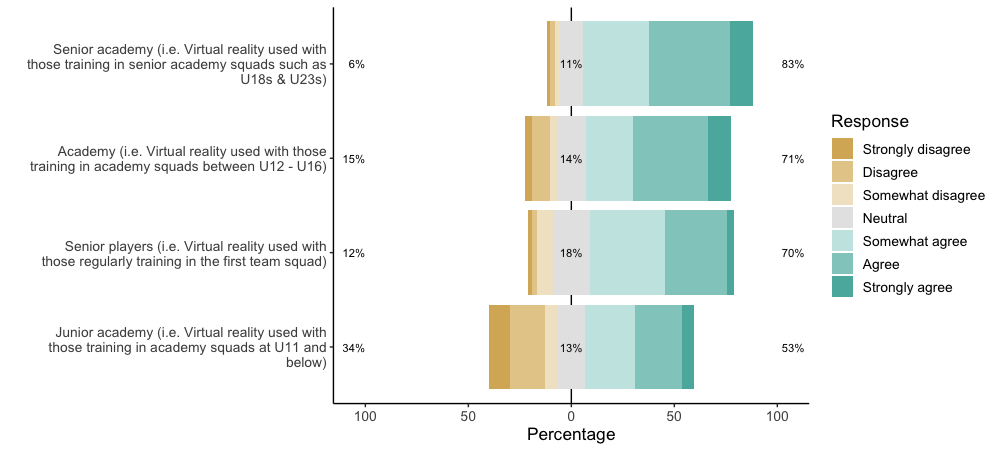

Supplement: S1 Fig — Likert bar-plot of responses by coaches/support staff to statements regarding who VR should be used with. Percentages indicate overall disagreement, neutral and overall agreement, from left to right respectively. (TIFF) [file pone.0261378.s001.tiff]
