# Supplementary material for: Perceptions of professional soccer coaches, support staff and players toward virtual reality and the factors that modify their intention to use it
Source: PLoS One. 2021 Dec 30;16(12):e0261378. doi: 10.1371/journal.pone.0261378 (PMC8717979; doi:10.1371/journal.pone.0261378)
Supplement: S1 Survey — (PDF) [file pone.0261378.s002.pdf]

The use of virtual reality in professional football

Introduction

Dear coach/support staff,

The purpose of contacting you is to ask if you would be willing to contribute to a research study that we are conducting. The project aims to examine and quantify the understanding and use of virtual reality within professional football. The online survey will enable us to examine coaches and support staff perspectives of their acceptance of technology, alongside their knowledge, expectations and potential barriers of using virtual reality within professional football training environments.

The survey consists of 8 sections (Section 1: General information; Section 2: Technology acceptance; Section 3: Knowledge of virtual reality; Section 4: Performance expectancy; Section 5: Social influence; Section 6: Using virtual reality; Section 7: Facilitating conditions; Section 8: Behavioural intention to use virtual reality)

Your completion of this survey will help us to ensure that our work is of practical relevance to people like yourself, and in return we will provide you with details of our research findings on completion.

Only the research team will have access to information disclosed in Section 1 (General information). In accordance with the 2018 General Data Protection Regulation, all information will remain anonymous and confidential to all co-researchers and will only be used as a point of reference when analysing the data. The survey will ask for the country your club is affiliated to, to understand the spread of the data, but this will not be

published. All documentation will be kept securely at the University of Hull.

The survey should be completed in one sitting and will take approximately 10 minutes. You will not be able to save and return to the survey at a later date so please consider this before starting. Data from this survey will only be submitted when clicking the finish button on the final page. No data will be collected whilst taking part in the survey and you will able to exit the survey at any point by closing the browser or survey tab. Should you wish to withdraw your data after submission, you will be allowed to do so by contacting any of the research team up until 01/05/2020. When contacting please provide your unique reference number which will be provided on submission of the survey. Withdrawal requests made without this number will not be possible due to the data being anonymous and therefore individual responses can't be identified. Any withdrawal requests after the 02/05/2020 might not be possible due to data analysis having already taken place.

If at any time you have questions related to the survey, please contact any of the supervisory team using the details found below.

Thank you in advance for taking the time to complete this survey.

Kind regards,

Ben Greenhough
PhD candidate t: 07909202620
The University of Hull e: B.Greenhough-2018@hull.ac.uk

Dr Grant Abt t: 01482 463397
The University of Hull e: g.abt@hull.ac.uk

Dr Chris McLaren-Towlson t: 01482 466047
The University of Hull e: c.towlson@hull.ac.uk

Dr Steve Barrett
Playermaker e: steve.barrett@playermaker.com

By clicking on the 'yes' button below you agree that you have:

* Read and understood all the information provided above.
* You understand that participation is voluntary and you're able to withdraw at any time.

Do you consent to taking part?

○ Yes ○ No

Qualification question

Do you currently work in a professional football club 'OR' a national association team?

- ○ Yes
- ○ No

Qualification question

Are you 17 years of age or older?

- ◯ Yes
- ◯ No

Qualification question

Have you completed and submitted this survey previously?

- Yes
- No

Section 1: General information

This section refers to your general details and background information as an individual working within a professional football club/team.

Section 1: General information

What gender do you identify with?

- Male
- Female
- Prefer not to say
- Other (no explanation necessary)
- Prefer to self describe

If you selected prefer to self describe, please specify:

What is your age (in years)?

- 17-21
- 22-26
- 27-31
- 32-36
- 37-41
- 42-46
- 47-51
- 52+

Which gender of football player do you primarily work with?

- Male
- Female

What age of players' do you primarily work with?

- Senior players (1st team)
- Senior academy (U18s/U23s)
- Academy (U12s-U16s)
- Junior academy (U11s and below)

What country is your club/team affiliated to? *This data will not be published.*

- England
- Scotland
- Wales
- Northern Ireland
- Republic of Ireland
- United States of America
- Australia
- Other

If you selected Other, please specify:

What tier of football pyramid does the first team within your club compete in? E.g. In England, a tier 1 team would be one that competes in the Premier League and a tier 2 team would be one that competes in the EFL Championship. If you only work for a national association team, select 'National football assocation team'.

- Tier 1
- Tier 2
- Tier 3

- Tier 4
- Tier 5
- National football association team

What is your primary role in the club/team?

- Manager/Head coach
- Technical coach
- Physiotherapist
- Sports scientist
- Strength and conditioning coach
- Rehabilitation coach
- Performance analyst
- Medical doctor
- Other

If you selected Other, please specify:

What is the highest academic qualification you have obtained?

- Doctorate (PhD)
- Masters (MSc/MRes/MPhil)
- Bachelors (BSc/BA)
- Foundation Degree
- Higher National Diploma (HND)
- Certificate of Higher Education

- High School Diploma
- GCSEs
- NVQ
- Business and Technology Education Council (BTEC)
- None
- Other

If you selected Other, please specify:

Section 2: Technology acceptance

This section refers to your general view on technology and how it has an influence on your life.

Section 2: Technology acceptance

We are interested in your views on how technology influences your life. Please indicate how much you agree with the following statements.

Technology gives me more freedom of mobility

- Strongly disagree
- Somewhat disagree
- Neutral
- Somewhat agree
- Strongly agree

Technology makes me more productive in my personal life

- Strongly disagree
- Somewhat disagree
- Neutral
- Somewhat agree
- Strongly agree

Other people come to me for advice on new technologies

- Strongly disagree
- Somewhat disagree
- Neutral
- Somewhat agree
- Strongly agree

In general, I am among the first in my circle of friends to acquire new technology when it appears

- Strongly disagree
- Somewhat disagree
- Neutral
- Somewhat agree
- Strongly agree

I keep up with the latest technological developments in my areas of interest

- Strongly disagree
- Somewhat disagree
- Neutral
- Somewhat agree
- Strongly agree

Technical support lines are not helpful because they don't explain things in terms I understand

- Strongly disagree
- Somewhat disagree
- Neutral
- Somewhat agree
- Strongly agree

Sometimes, I think that technology systems are not designed for use by ordinary people

- Strongly disagree
- Somewhat disagree
- Neutral
- Somewhat agree
- Strongly agree

People are too dependent on technology to do things for them

- Strongly disagree
- Somewhat disagree
- Neutral
- Somewhat agree
- Strongly agree

Too much technology distracts people to a point that is harmful

- Strongly disagree
- Somewhat disagree
- Neutral
- Somewhat agree
- Strongly agree

Technology lowers the quality of relationships by reducing personal interaction

- Strongly disagree
- Somewhat disagree
- Neutral
- Somewhat agree
- Strongly agree

“These questions comprise the Technology Readiness Index 2.0 which is copyrighted by A. Parasuraman and Rockbridge Associates, Inc., 2014. This scale may be duplicated only with written permission from the authors.”

Section 3: Knowledge of virtual reality

This section refers to your general knowledge of virtual reality for personal and professional use.

For the duration of this survey, virtual reality is defined as including a headset worn by a user that covers their eyes, allowing them to experience a virtual world that is created by a computer (**Image A**). This definition does not refer to non-immersive technology that results in an output through a television or other electronic interface (**Image B**). Our definition does not include augmented reality, whereby computer generated images are placed into the real world and viewed live (**Image C**).

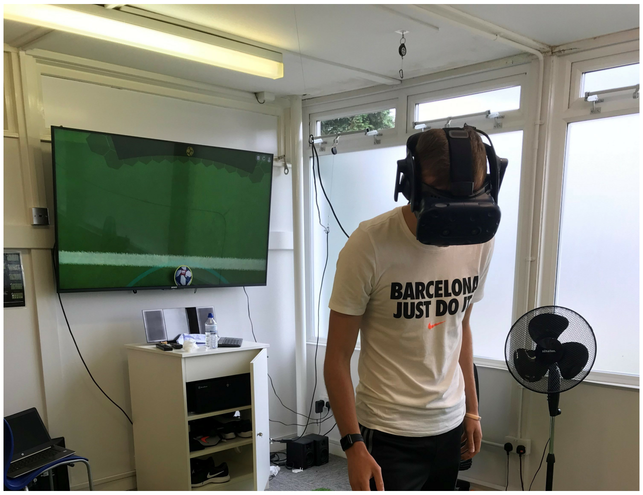

(A) Virtual reality headset

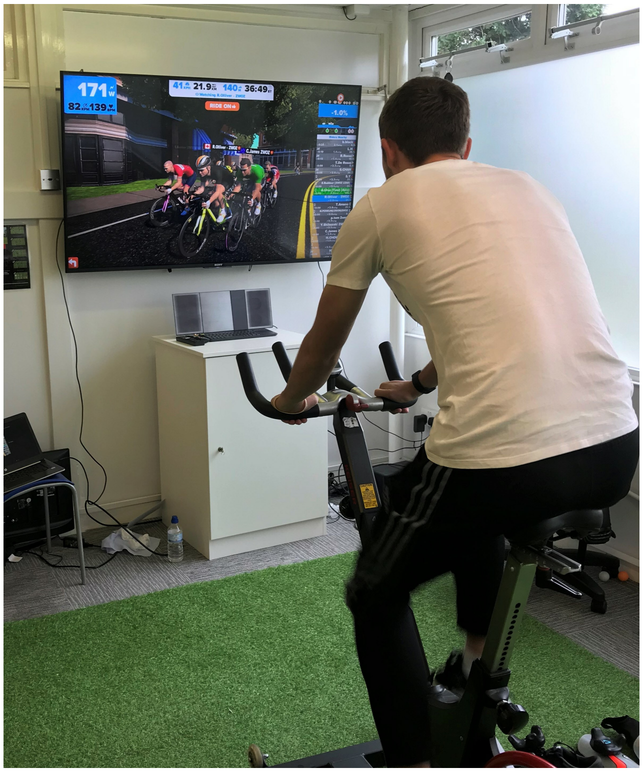

(B) Non-immersive virtual reality

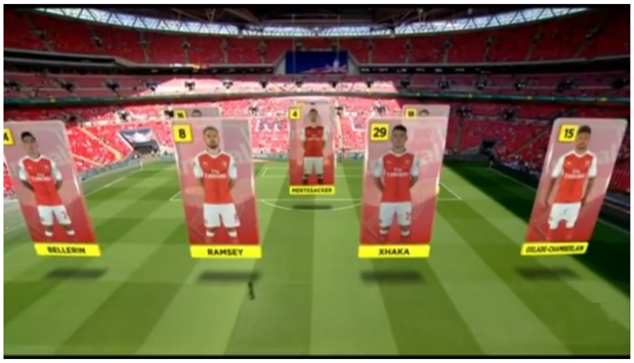

(C) Augmented reality

Section 3: Knowledge of virtual reality

Based on the definition on the previous slide, have you ever heard of or used a virtual reality system?

- ○ Yes
- ○ No

Are you aware of virtual reality being used within sport by coaches, support staff or athletes? (your answer should be based on your knowledge prior to seeing this survey)

- ○ Yes
- ○ No

In which sports are you aware that virtual reality is being used by coaches, support staff or athletes?

- ☐ Football
- ☐ Rugby union
- ☐ Rugby league
- ☐ Cricket
- ☐ American Football
- ☐ Baseball
- ☐ Skiing
- ☐ Golf
- ☐ Not sure
- ☐ Other

If you selected Other, please specify:

For the remainder of the survey, questions related to the use or knowledge of virtual reality will be in the context of:

> *"Virtual reality used by coaches, support staff and players as part of training or personal use within professional football training grounds"*

Are you aware of virtual reality being used within professional football?

- Yes
- No

Have you used virtual reality within a professional football club? *If you have used virtual reality as part of a demonstration or trial, please answer 'yes'.*

- Yes
- No

When was your last experience of using virtual reality?

- Within the last year
- Between 1 - 2 years ago
- Between 3 - 5 years ago
- Between 6 - 10 years ago
- Longer than 11 years ago

Section 4: Performance expectancy

The next section refers to your expectations of virtual reality being able to improve performance and wellbeing .

Section 4: Performance expectancy

For the questions below, base your answers on the group of players you primarily work with.

State your level of agreement (strongly disagree - strongly agree) on your expectations of virtual reality being able to improve the following areas:

Physical fitness (i.e. Virtual reality used with players to improve areas such as strength, power, aerobic fitness etc.)

- Strongly disagree
- Disagree
- Somewhat disagree
- Neither agree or disagree
- Somewhat agree
- Agree
- Strongly agree

Cognition (i.e. Virtual reality used with players to improve cognition such as decision making, reaction time, visual awareness etc.)

- Strongly disagree
- Disagree
- Somewhat disagree
- Neither agree or disagree
- Somewhat agree
- Agree
- Strongly agree

Technical skill (i.e. Virtual reality used with players to improve technical ability such as passing & shooting accuracy etc.)

- Strongly disagree
- Disagree
- Somewhat disagree
- Neither agree or disagree
- Somewhat agree
- Agree
- Strongly agree

Tactical development (i.e. Virtual reality used with players to improve awareness of team tactics etc.)

- Strongly disagree
- Disagree
- Somewhat disagree
- Neither agree or disagree
- Somewhat agree
- Agree
- Strongly agree

Mental wellbeing (i.e. Virtual reality used with players to improve mental wellbeing such as stress and anxiety etc.)

- Strongly disagree
- Disagree
- Somewhat disagree
- Neither agree or disagree

- Somewhat agree
- Agree
- Strongly agree

Performance expectancy

For the questions below, base your answers on the group of players you primarily work with.

State your level of agreement (strongly disagree - strongly agree) for virtual reality to be used in the following applications:

Training tool (i.e. Virtual reality used as part of training sessions - pre-activation, warm ups, skill development etc.)

- Strongly disagree
- Disagree
- Somewhat disagree
- Neither agree or disagree
- Somewhat agree
- Agree
- Strongly agree

Testing tool (i.e. Virtual reality used as a testing tool to inform training practice and set benchmarks)

- Strongly disagree
- Disagree
- Somewhat disagree
- Neither agree or disagree
- Somewhat agree

- Agree
- Strongly agree

Monitoring tool (i.e. Virtual reality used as a monitoring tool to assess readiness to train)

- Strongly disagree
- Disagree
- Somewhat disagree
- Neither agree or disagree
- Somewhat agree
- Agree
- Strongly agree

Performance analysis (i.e. Virtual reality used to view simulated footage from the view of the player, coach or different view points within the stadium)

- Strongly disagree
- Disagree
- Somewhat disagree
- Neither agree or disagree
- Somewhat agree
- Agree
- Strongly agree

Preparation tool (i.e. Virtual reality used to prepare players for unfamiliar environments such as placing them within a stadium they've never played in

previously)

- Strongly disagree
- Disagree
- Somewhat disagree
- Neither agree or disagree
- Somewhat agree
- Agree
- Strongly agree

Rehabilitation (i.e. Virtual reality used with players undergoing rehabilitation)

- Strongly disagree
- Disagree
- Somewhat disagree
- Neither agree or disagree
- Somewhat agree
- Agree
- Strongly agree

Talent identification tool (i.e. Virtual reality used to implement talent identification protocols)

- Strongly disagree
- Disagree
- Somewhat disagree
- Neither agree or disagree
- Somewhat agree
- Agree

- Strongly agree

Performance expectancy

State your level of agreement (Strongly disagree - Strongly agree) for virtual reality to be used with the following age groups of players:

Senior players (i.e. Virtual reality used with those regularly training in the first team squad)

- Strongly disagree
- Disagree
- Somewhat disagree
- Neither agree or disagree
- Somewhat agree
- Agree
- Strongly agree

Senior academy (i.e. Virtual reality used with those training in senior academy squads such as U18s & U23s)

- Strongly disagree
- Disagree
- Somewhat disagree
- Neither agree or disagree
- Somewhat agree
- Agree
- Strongly agree

Academy (i.e. Virtual reality used with those training in academy squads between U12 - U16)

- Strongly disagree
- Disagree
- Somewhat disagree
- Neither agree or disagree
- Somewhat agree
- Agree
- Strongly agree

Junior academy (i.e. Virtual reality used with those training in academy squads at U11 and below)

- Strongly disagree
- Disagree
- Somewhat disagree
- Neither agree or disagree
- Somewhat agree
- Agree
- Strongly agree

Section 5: Social influence

The next section refers to social factors that could influence your decision to use virtual reality technology.

Section 5: Social influence

Using the statements below, please indicate how you might be socially influenced (not at all influenced - highly influenced) to use virtual reality within a professional football environment.

To be seen using an innovative technology (i.e. I would be influenced to use virtual reality so that others see me using an innovative technology)

- Not at all influenced
- Slightly influenced
- Somewhat influenced
- Very influenced
- Extremely influenced

Influential others use virtual reality (i.e. I would be influenced to use virtual reality if individuals that influence me also use it)

- Not at all influenced
- Slightly influenced
- Somewhat influenced
- Very influenced
- Extremely influenced

Influential clubs use virtual reality (i.e. I would be influenced to use virtual reality if clubs that influence me also use it)

- Not at all influenced
- Slightly influenced

- Somewhat influenced
- Very influenced
- Extremely influenced

Those senior to me (i.e. I would be influenced to use virtual reality if individuals that are senior to me want it to be used)

- Not at all influenced
- Slightly influenced
- Somewhat influenced
- Very influenced
- Extremely influenced

Player enjoyment (i.e. I would be influenced to use virtual reality if players enjoyed using the system)

- Not at all influenced
- Slightly influenced
- Somewhat influenced
- Very influenced
- Extremely influenced

Virtual reality access

Do the group of players that you primarily work with in your club have access to virtual reality within the training ground facilities? *If your club has virtual reality but the players you work with don't have access to it (e.g, available only for use by the senior team), click No. If you're not sure if your club has access it it, click No.* ✱ *Required*

- ○ Yes
- ○ No

Section 6: Use of virtual reality

The next section refers to how virtual reality is currently being used within your club, or how you would look to use it within your club.

Section 6: Use of virtual reality

Answer the following questions based on how you use virtual reality within the club you work for.

Are you directly responsible for operating and/or instructing players through virtual reality based acitivites?

- ◯ Yes
- ◯ No

Which of the following do you use virtual reality for? *Select all that apply*

- ☐ Training
- ☐ Testing
- ☐ Monitoring
- ☐ Performance analysis
- ☐ Entertainment
- ☐ Rehabilitation
- ☐ Other

If you selected Other, please specify:

For each player, how long would you typically use virtual reality for?

- ◯ Less than 5 minutes
- ◯ 5-10 minutes
- ◯ 11-20 minutes
- ◯ 21-30 minutes

- 31 minutes +

How frequently do you use virtual reality within your club?

- Every day
- A few times a week
- Once a week
- A few times a month
- Once a month
- Less than once a month
- Never

Which of the following do you aim to change by using virtual reality? *If you use virtual reality for a specific purpose that is not mentioned below, please select 'Other'. Select all that apply.*

- [ ] Physical fitness
- [ ] Cognition
- [ ] Technical skill
- [ ] Tactical awareness
- [ ] Mental well-being
- [ ] Other

If you selected Other, please specify:

How long has your club had access to virtual reality? ✱ *Required*

- Less than 6 months

- 6-12 months
- 1-2 years
- 3-4 years
- 5 years +
- Not sure

Pre-access perceptions of virtual reality

For the following questions we would like you to reflect on your opinions of using virtual reality in professional football both before and after having had access to it.

Before having access to virtual reality at your training ground, what was your overall opinion on virtual reality technology for use by coaches, support staff and players?

- Very negative
- Negative
- Neutral
- Positive
- Very positive
- Wasn't aware of its use within football training grounds

Since having access to virtual reality at your training ground, what is your overall opinion on virtual reality technology for use by coaches, support staff and players?

- Very negative
- Negative
- Neutral
- Positive

◯ Very positive

Section 7: Facilitating conditions

The next section refers to any perceived barriers to using virtual reality.

Section 7: Facilitating conditions

Please indicate how much each of the following is a barrier (Not a barrier - Extreme barrier) to continual use of virtual reality within your club/team:

Note: If you have access and are currently using virtual reality with your players, answer in the context of 'continuing to use it (virtual reality)'

Are you roughly aware of the cost of having access to virtual reality within the training ground facilities?

- Yes
- No

Cost (i.e. The cost of virtual reality is a barrier to its continual implementation)

- Not a barrier
- Somewhat a barrier
- Moderate barrier
- Extreme barrier

Player buy-in (i.e. Getting players to engage with the virtual reality system is a barrier to using it)

- Not a barrier
- Somewhat a barrier
- Moderate barrier
- Extreme barrier

Coach and support staff buy-in (i.e. Coaching staff buy-in to virtual reality being used with players is a barrier to using it)

- Not a barrier
- Somewhat a barrier
- Moderate barrier
- Extreme barrier

Personnel to operate (i.e. requiring personnel to operate the virtual reality system is a barrier to using it)

- Not a barrier
- Somewhat a barrier
- Moderate barrier
- Extreme barrier

Space to operate (i.e. space within the training ground to operate the virtual reality system is a barrier to using it)

- Not a barrier
- Somewhat a barrier
- Moderate barrier
- Extreme barrier

Limited evidence base (i.e. limited research available on virtual reality used in professional football is a barrier to using it)

- Not a barrier

- Somewhat a barrier
- Moderate barrier
- Extreme barrier

Time available (i.e. time available to use within schedule is a barrier to using virtual reality)

- Not a barrier
- Somewhat a barrier
- Moderate barrier
- Extreme barrier

First impression (i.e. my first impression of using, seeing or hearing about virtual reality is a barrier to using it)

- Not a barrier
- Somewhat a barrier
- Moderate barrier
- Extreme barrier

Section 7: Facilitating conditions

The next section refers to your perceived barriers to using virtual reality.

Section 7: Facilitating conditions

Please state your perceived barriers (Not a barrier - Extreme barrier) to using virtual reality within your club or your group of players:

Are you roughly aware of the cost of having access to virtual reality within the training ground facilities?

- Yes
- No

Cost (i.e. The cost of virtual reality is a barrier to its implementation)

- Not a barrier
- Somewhat a barrier
- Moderate barrier
- Extreme barrier

Player buy-in (i.e. I envisage getting players to engage with the virtual reality system is a barrier to using it)

- Not a barrier
- Somewhat a barrier
- Moderate barrier
- Extreme barrier

Coach and support staff buy-in (i.e. I envisage coaching staff buy-in to virtual reality being used with players is a barrier)

- Not a barrier
- Somewhat a barrier
- Moderate barrier
- Extreme barrier

Personnel to operate (i.e. requiring personnel to operate the virtual reality system is a barrier to using it)

- Not a barrier
- Somewhat a barrier
- Moderate barrier
- Extreme barrier

Space to operate (i.e. space within the training ground to operate the virtual reality system is a barrier to using it)

- Not a barrier
- Somewhat a barrier
- Moderate barrier
- Extreme barrier

Limited evidence base (i.e. limited research available on virtual reality used in professional football is a barrier to using it)

- Not a barrier
- Somewhat a barrier
- Moderate barrier

- Extreme barrier

Time available (i.e. time available within schedule is a barrier to using virtual reality)

- Not a barrier
- Somewhat a barrier
- Moderate barrier
- Extreme barrier

First impression (i.e. my first impression of using, seeing or hearing about virtual reality is a barrier to using it)

- Not a barrier
- Somewhat a barrier
- Moderate barrier
- Extreme barrier

Section 8: Intention to use virtual reality

The last section refers to your intention to use virtual reality in the future.

Section 8: Intention to use virtual reality

Have the group of players that you primarily work with in your club, previously had access to virtual reality within the training ground facilities?

- Yes
- No

If virtual reality technology was made available to you, how likely are you to use it within your club?

- Extremely unlikely
- Unlikely
- Neutral
- Likely
- Extremely likely

What is your overall opinion of virtual reality technology for use by coaches, support staff and players within the training ground setting?

- Very negative
- Negative
- Neutral
- Positive
- Very positive

Final page

Thank you for taking the time to complete this survey. If you have any questions or you would like withdraw your consent then please contact one of the research team listed below, referencing the receipt number provided above. Failure to provide this number will not allow the research team to discuss your submission or withdraw your data.

Ben Greenhough
PhD candidate t: 07909202620
The University of Hull e: B.Greenhough-2018@hull.ac.uk

Dr Grant Abt t: 01482 463397
The University of Hull e: g.abt@hull.ac.uk

Dr Chris McLaren-Towlson t: 01482 466047
The University of Hull e: c.towlson@hull.ac.uk

Dr Steve Barrett
Hull City Football Club e: steve.barrett@hulltigers.com
