# Supplementary material for: Perceptions of professional soccer coaches, support staff and players toward virtual reality and the factors that modify their intention to use it
Source: PLoS One. 2021 Dec 30;16(12):e0261378. doi: 10.1371/journal.pone.0261378 (PMC8717979; doi:10.1371/journal.pone.0261378)
Supplement: S1 Table — (DOCX) [file pone.0261378.s004.docx]

**S1 Table. *Proportion and frequency of coach/support staff and player responses to awareness and experience of using VR.***

| **Question** | **Answer** | **Practitioner % (n)** | **Player % (n)** |
| --- | --- | --- | --- |
| **Have you ever heard of, or used a virtual reality system?** | Yes | 94% (134) | 89% (57) |
|  | No | 6% (9) | 11% (7) |
| **Are you aware of VR in professional soccer** | Yes | 76% (108) | 72% (46) |
|  | No | 24% (35) | 28% (18) |
| **Have you used virtual reality within a professional soccer club? If you have used virtual reality as part of a demonstration or trial, please answer 'yes'.** | Yes | 30% (43) | 47% (30) |
|  | No | 70% (100) | 53% (34) |
| **When was your last experience of using virtual reality within a professional soccer club?** | Never used | 70% (100) | 53% (34) |
|  | Within the last year | 22% (32) | 44% (28) |
|  | Between 1 - 2 years ago | 6% (8) | 3% (2) |
|  | Between 3 - 5 years ago | 1% (2) |  |
|  | Between 6 - 10 years ago | 1% (1) |  |
