# Supplementary material for: Perceptions of professional soccer coaches, support staff and players toward virtual reality and the factors that modify their intention to use it
Source: PLoS One. 2021 Dec 30;16(12):e0261378. doi: 10.1371/journal.pone.0261378 (PMC8717979; doi:10.1371/journal.pone.0261378)
Supplement: Attachment — Submitted filename: Response to Reviewers.docx [file pone.0261378.s005.docx]

**Response to the reviewers**

Dear editor and reviewers,

We would like to thank you for taking the time to read our manuscript and providing us with useful comments so we can strengthen our work.

Below I have included each comment made by the editor and reviewers and indicated our response to each comment, highlighted in red.

We believe that this manuscript is now suitable for publication with PLOS ONE.

Yours sincerely,

Benjamin Greenhough

**Editor comments**

1. Please ensure that your manuscript meets PLOS ONE's style requirements, including those for file naming. The PLOS ONE style templates can be found at

<https://journals.plos.org/plosone/s/file?id=wjVg/PLOSOne_formatting_sample_main_body.pdf>

<https://journals.plos.org/plosone/s/file?id=ba62/PLOSOne_formatting_sample_title_authors_affiliations.pdf>

Edits have been made to the manuscript to meet the PLOS ONE style requirements as documented in the links above.

2. Please include additional information regarding the survey or questionnaire used in the study and ensure that you have provided sufficient details that others could replicate the analyses. For instance, if you developed a questionnaire as part of this study and it is not under a copyright more restrictive than CC-BY, please include a copy, in both the original language and English, as Supporting Information.

Both surveys for coaches/support staff and players have been included in the supporting information as PDF files.

3. Please upload a copy of Supporting Information “S2 Text. Surveys’ definition of virtual reality” which you refer to in your text on page 24.

I have subsequently removed “S2 Text. Survey definition of virtual reality” based on the comments I made back to reviewer 1 (see below).

4. Please review your reference list to ensure that it is complete and correct. If you have cited papers that have been retracted, please include the rationale for doing so in the manuscript text, or remove these references and replace them with relevant current references. Any changes to the reference list should be mentioned in the rebuttal letter that accompanies your revised manuscript. If you need to cite a retracted article, indicate the article’s retracted status in the References list and also include a citation and full reference for the retraction notice.

Reference list has been checked to ensure it is complete and correct. One new reference has been added (reference 30) to answer reviewer 2 comment. Reference has been included below

**Reviewer comments 1**

After reading the manuscript over a few times, I feel that my comments are very generic guidelines that specific sentences or paragraphs. The authors are welcome to adjust their manuscript accordingly, or disagree with my comments too. As long as they are justified from the authors’ perspective.

1. The authors appear to be targeting this manuscript to staff in high-performance, but from my experience unfortunately I can imagine that they will most likely lose attention throughout reading the text. I understand that many of the data is necessary with the use of such questionnaire, but 6 tables, 5 figures and 6 supplementary files was a bit overwhelming. Are all these necessary including all of the text describing the findings too? It just seems that the manuscript only states facts, whereas there is not so much "why" attached to a lot of the "what".

   We do agree with the reviewer’s comments. The manuscript is lengthy, with a lot of figures and tables. That said, this comment conflicts with reviewer 2’s comments, where they suggest adding two more figures below (diagrams of the SEM). As such, we have removed some supplementary information (S2, S3 and S4) as they are captured in the surveys (S5 and S5). We have removed the demographic text (original text lines 290-294) which is already captured in table 2. Table 3 has been moved to the supplementary materials (as its use in the manuscript is limited). We have also condensed the text between lines 311 – 364 (original text) to just a few of the keys findings, as this text is a repeat of what is shown in each figure. We have just added one of the SEM models (picture included for your reference at bottom of this file) that reviewer two has suggested.
2. This comment is once more related to the target audience of this manuscript. Is it possible that with the word count potentially shortened for the next draft that the authors could include a practical application/recommendations paragraph for practitioners who want to implement/improve adoption rate for VR into their club? For example, the authors could explain that focusing on the social influences of VR to get teams to use it is not the right approach (whereas the media department might favour this), but instead constructing a clear argument of VR for stakeholders in the club i) to support the physios training injured players with supported by recent research (Gokeler), ii) to support performance analysis department for tactical analysis and iii) collaborations with universities with VR technology that would loan out headsets to clubs in exchange for data – thereby reducing the cost barrier which directly solves the largest implementation barrier of VR.

We have included a practical applications paragraph after the conclusions, which does help to summarise some of the take-home messages of the manuscript. Thank you for the suggestion.

3. One small thing: The title is slightly misrepresenting of the population of the sample. I wouldn’t use coaches and players, as in the manuscript there are many other support staff that are not coaches. I believe you’ve referred to them collectively as “practitioners” in L143.

We have included ‘support staff’ in the title to represent a large proportion of the sample that are not coaches. We then refer to them as practitioners in the methods and then throughout the manuscript to allow for better reading.

**Reviewer 2 comments**

The ethics statement mentions Declaration of Helsinki, but the latest version of this (item 35 in https://www.wma.net/policies-post/wma-declaration-of-helsinki-ethical-principles-for-medical-research-involving-human-subjects/) requires preregistration. As far as I can tell, this was not done by the authors, so they should at least mention which version of the Declaration their study adhered to (or remove this altogether). (Also on line 140-141.)

Thank you for providing this information. We weren’t aware of the need to preregister the study to include the Declaration of Helsinki. This study is not preregistered and so we have removed this part of the ethics statement.

Abstract could be more informative about the findings and its implications.

We feel that the abstract adequately conveys the aim, method, results summary, and main implications. However, we have made some minor adjustments to aid clarity.

Introduction: very clear motivation for the study – nothing to comment, really.

Thank you

Line 165-167: Do you mean screen-based VR (e.g., CAVE-like set-ups) was not included? That needs to be made explicit throughout.

Yes, CAVE-like set ups were not included as part of our definition of VR. We have included a sentence on line 186 to make this explicit, along with a reference for readers to learn more about CAVE set ups.

Line 189: a visual representation of the model would make sense for the less informed readers.

We have included a visual representation of the model in Figure 6 (line 485).

Power calculation/interpretation of p-values: was the amount of effects included in the model considered? Any regression based model with multiple predictor variables is influenced by issues of multiple comparison just as e.g., when performing multiple t-tests. Using alpha = 0.05 for individual predictor variables thus increases the chance of erroneously including these in the model (Type 1 errors). I would expect some treatment of this issue in the paper. See e.g., <http://citeseerx.ist.psu.edu/viewdoc/download?doi=10.1.1.490.7640&rep=rep1&type=pdf>

As outlined by Henseler et al (2014) the bias-corrected and accelerated confidence intervals (BCa) bootstrapping method as implemented in SmartPLS maintains the type 1 error rate at 5%. We have added that clarification to line 275-278.

Henseler, J., Dijkstra, T. K., Sarstedt, M., Ringle, C. M., Diamantopoulos, A., Straub, D. W., Ketchen, D. J., Hair, J. F., Hult, G. T. M., & Calantone, R. J. (2014). Common Beliefs and Reality About PLS. Organizational Research Methods, 17(2), 182–209. https://doi.org/10.1177/1094428114526928

Line 230: how was this non-normality dealt with? Was normality not an assumption of the statistical model? This is important information since it could affect the validity of the statistical results/inferences.

Normality is not an assumption with PLS-SEM. We have included a sentence and reference within the revised manuscript (line 251).

Line 260: ‘strong theoretical support’ sounds somewhat vague.

We have edited this sentence from “required strong theoretical support for its inclusion in the formative construct model” to required (1) a rationale for its inclusion in the formative construct model through either anecdotal or empirical evidence [29], and (2) removing the indicator variable doesn’t change the conceptual meaning of the construct [30] (lines 286-288).

The paper makes a distinction between potential cognitive, mental, tactical, physical, and technical benefits of VR. I think this needs to be defined very clearly in the Introduction. I would argue that a potential benefit (sensorimotor/visuomotor control, or something of this nature) is not perfectly captured by any of these terms, although I suspect the authors might capture this under ‘cognitive’ (which I think is suboptimal). Virtual reality could have training benefits in terms of an improved link between perception and action (e.g., better ability to link visual information about the kicker’s kinematics and ball flight in free kick or penalty scenarios to actions through VR training using a large database of kick kinematics).

In the Introduction we already refer to perceptual motor-skills (line 116), and studies related to this. We feel that this clearly highlights to the reader that perception/action is a potential benefit of VR.

Some readers may prefer a visual representation of the SEM – there are standardized ways to present these. In general, the current presentation of the model results is hard to follow (in terms of the interpretation/meaning of the results).

We have now added a figure (Figure 6) to visually represent the SEM model (line 485).

The discussion refers to the names of the factors in the model (‘performance expectancy’, ‘social influence’ etc), which sometimes affects readability. I would suggest writing out these in terms of the actual meaning to help the reader along. For example

“Our study allows us to conclude that performance expectancy is the largest contributor towards likeliness to use VR in professional soccer.”

->

“Our study allows us to conclude that likeliness to use VR in professional soccer mostly depends on

expectations concerning performance benefits of using VR.”

Thank you for that suggestion. We have made edits to the discussion/conclusion to improve the readability.
